# Supplementary figures and images for: Genetic evidence for a fall-spawning group of Gulf sturgeon (Acipenser oxyrinchus desotoi) in the Apalachicola River, Florida, USA
Source: PLoS One. 2025 Jun 3;20(6):e0319117. doi: 10.1371/journal.pone.0319117 (PMC12132950; doi:10.1371/journal.pone.0319117)

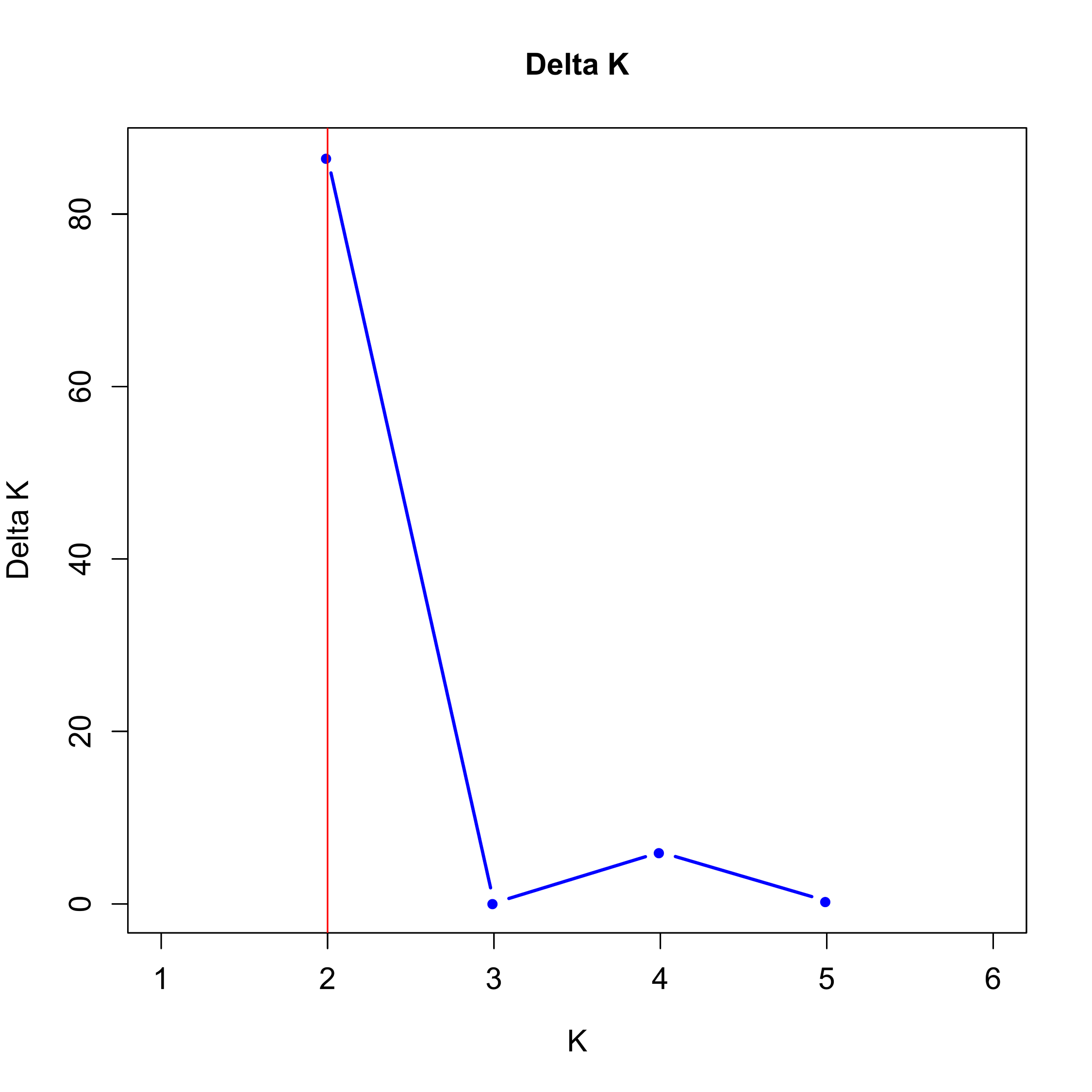

Supplement: S1 Fig — All samples were ≤520 mm FL and collected from 2013–2022. To identify the most likely number of genetic groups, ΔK analysis determines the rate of change in log likelihood of the STRUCTURE data between each value of K. (TIF) [file pone.0319117.s003.tif]
