# Supplementary material for: Genetic evidence for a fall-spawning group of Gulf sturgeon (Acipenser oxyrinchus desotoi) in the Apalachicola River, Florida, USA
Source: PLoS One. 2025 Jun 3;20(6):e0319117. doi: 10.1371/journal.pone.0319117 (PMC12132950; doi:10.1371/journal.pone.0319117)
Supplement: Attachment — Submitted filename: Reviewer Attachments for PONE-D-25-04780.docx [file pone.0319117.s004.docx]

Genetic evidence for a fall spawning group of Gulf sturgeon (*Acipenser oxyrinchus desotoi*) in the Apalachicola River, Florida

| 1. **Line No.** | **Section** | **Comments/Suggestions** |
| --- | --- | --- |
| **1-5** | **Title** | The title is clear and concise. Suggestion: Add "Florida, USA" for geographic specificity. |
| **19-44** | **Abstract** | The abstract provides a good overview. Suggestion: Briefly mention the genetic techniques used (e.g., microsatellite analysis) and the specific implications for conservation management. |
| **47-72** | **Introduction** | The background on Gulf sturgeon is solid. Suggestion: Briefly mention the genetic tools used in similar studies on other sturgeon populations (e.g., Atlantic sturgeon) to set the stage for the current study. |
| **73-100** | **Introduction** | The discussion of the Apalachicola River is well-detailed. Suggestion: Add a sentence or two about the potential genetic implications of the Jim Woodruff Lock and Dam on population structure. |
| **113-132** | **Methods (Sampling)** | Sampling methods are well-described. Suggestion: Include a brief justification for the specific mesh sizes used in the gill nets, as this could influence the size range of captured fish. |
| **133-155** | **Methods (Genetic Analysis)** | Genetic methods are clearly outlined. Suggestion: Add a brief explanation of why the specific microsatellite loci were chosen and their relevance to Gulf sturgeon. |
| **156-184** | **Methods (Statistical Analysis)** | Statistical methods are appropriate. Suggestion: Include a brief explanation of why STRUCTURE was chosen over other population structure analysis tools. |
| **193-198** | **Results (Sample Size)** | The exclusion of 45 fish is noted. Suggestion: Provide a brief explanation of why these exclusions were necessary (e.g., missing data, size thresholds). |
| **199-212** | **Results (STRUCTURE Analysis)** | The results of the STRUCTURE analysis are clearly presented. Suggestion: Add a brief discussion of the potential implications of the mixed ancestry individuals (q-scores < 0.70). |
| **220-237** | **Results (Length Frequency)** | Length frequency distributions are well-described. Suggestion: Include a brief discussion of the potential ecological or environmental factors driving the bimodal distribution in the fall-spawned group. |
| **275-282** | **Discussion (Fall Spawning)** | The discussion of fall spawning evidence is strong. Suggestion: Add a brief comparison with other rivers (e.g., Suwannee, Choctawhatchee) to provide broader context. |
| **294-313** | **Discussion (Recruitment)** | The discussion of recruitment success is insightful. Suggestion: Include a brief mention of potential environmental factors (e.g., temperature, flow) that may influence recruitment in the fall. |
| **314-322** | **Discussion (Genetic Diversity)** | The discussion of genetic diversity is well-presented. Suggestion: Add a brief mention of the potential long-term implications of lower genetic diversity in the fall-spawned group. |
| **323-349** | **Discussion (Management)** | The management implications are well-articulated. Suggestion: Include a brief discussion of how the findings could inform future dam operations or habitat restoration efforts. |
| **351-358** | **Acknowledgements** | The acknowledgements are appropriate and comprehensive. No changes needed. |
| **360-536** | **References** | The references are thorough and relevant. Suggestion: Add a few more recent studies on Gulf sturgeon genetics or fall spawning in other species for broader context. |
| **207-212** | **Figure 1** | The figure is clear and effectively illustrates the STRUCTURE analysis results. Suggestion: Add a brief caption explaining the significance of the gray and white bars. |
| **241-243** | **Figure 2** | The figure is well-presented. Suggestion: Include a brief caption explaining the significance of the bimodal distribution in the fall-spawned group. |
| **214-219** | **Table 1** | The table is clear and informative. Suggestion: Add a brief footnote explaining the statistical significance of the differences between groups. |
| **539-546** | **Supporting Information** | The supporting information is well-organized. Suggestion: Provide a brief explanation of how the data in S1 Table were used in the analysis. |
